# Supplementary material for: The dynamic recruitment of TRBP to neuronal membranes mediates dendritogenesis during development
Source: EMBO Rep. 2017 Dec 20;19(3):e44853. doi: 10.15252/embr.201744853 (PMC5835843; doi:10.15252/embr.201744853)
Supplement: Supplementary file 1 — Appendix [file EMBR-19-e44853-s001.pdf]

Table of Contents

Appendix Figures **2**

Appendix Figure Legends **6**

Figure S1: Validation of fractionation and imaging results 6

Figure S2: Representative images and raw colocalization values between TRBP and ER markers. 6

Figure S3: TRBP phosphorylation does not affect its sub-cellular distribution or binding to Dicer 6

Figure S4: BDNF stimulation leads to a transient increase in the expression of the BDNF 3'UTR dual-luciferase reporter (BDNF 3'UTR WT), but not the miR-16-5p-binding site mutant (BDNF 3'UTR Mut). 7

Appendix Table S1: Functional annotation clustering of miR-22-3p and miR-9a-3p isomiRs.. .... **8**

Appendix Figures

Figure S1

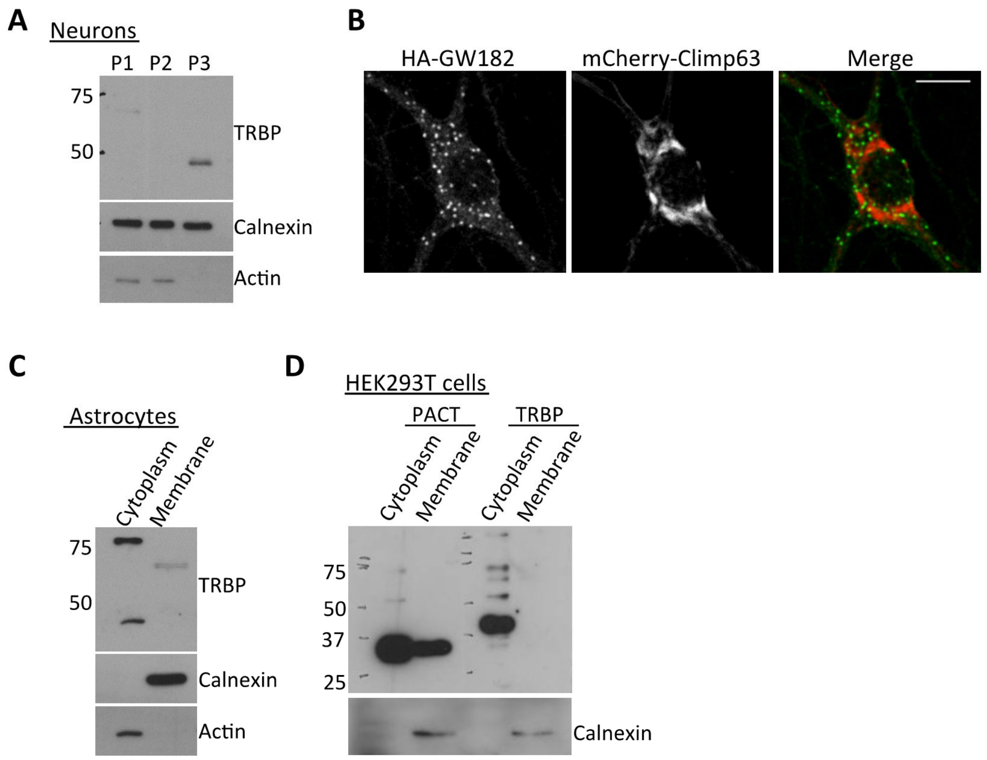

Figure S2

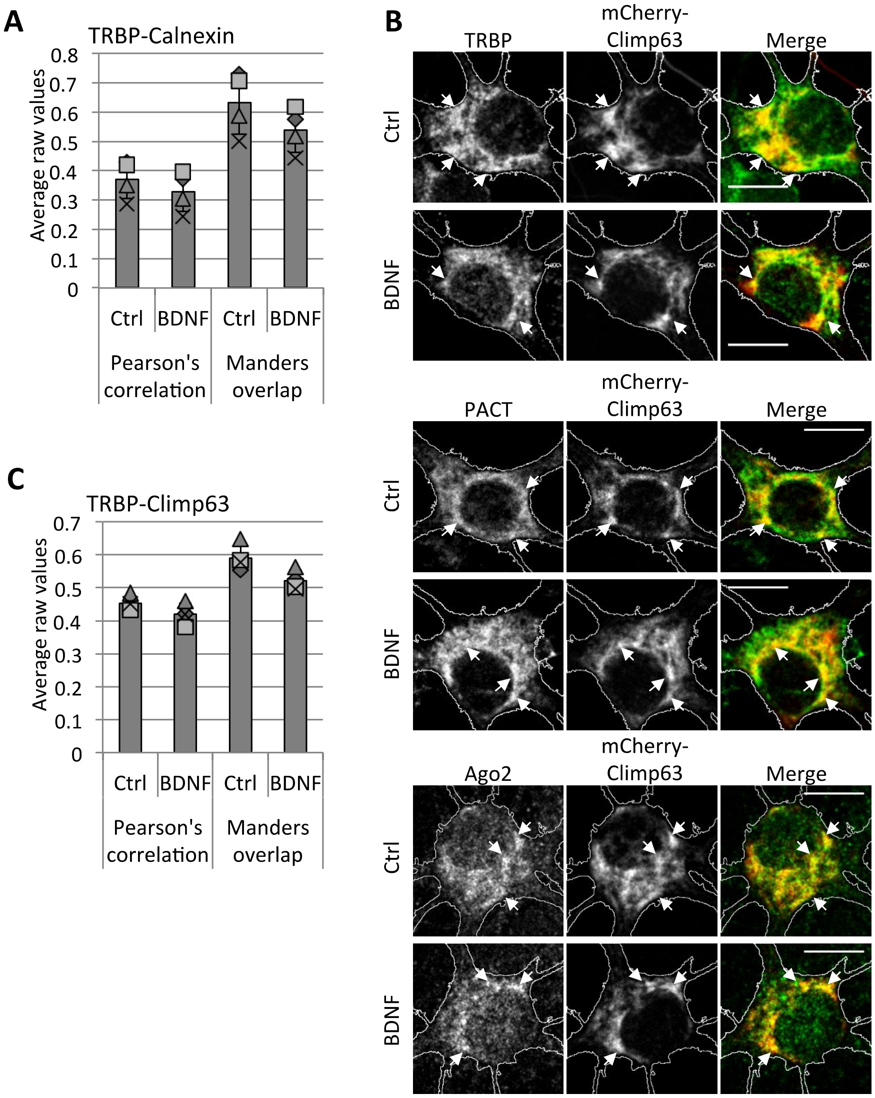
A
TRBP-Calnexin
Average raw values
0.8
0.7
0.6
0.5
0.4
0.3
0.2
0.1
0
Ctrl
BDNF
Ctrl
BDNF
Pearson's correlation
Manders overlap
B
TRBP
mCherry-Climp63
Merge
Ctrl
BDNF
PACT
mCherry-Climp63
Merge
Ctrl
BDNF
Ago2
mCherry-Climp63
Merge
Ctrl
BDNF
C
TRBP-Climp63
Average raw values
0.7
0.6
0.5
0.4
0.3
0.2
0.1
0
Ctrl
BDNF
Ctrl
BDNF
Pearson's correlation
Manders overlap

Figure S3

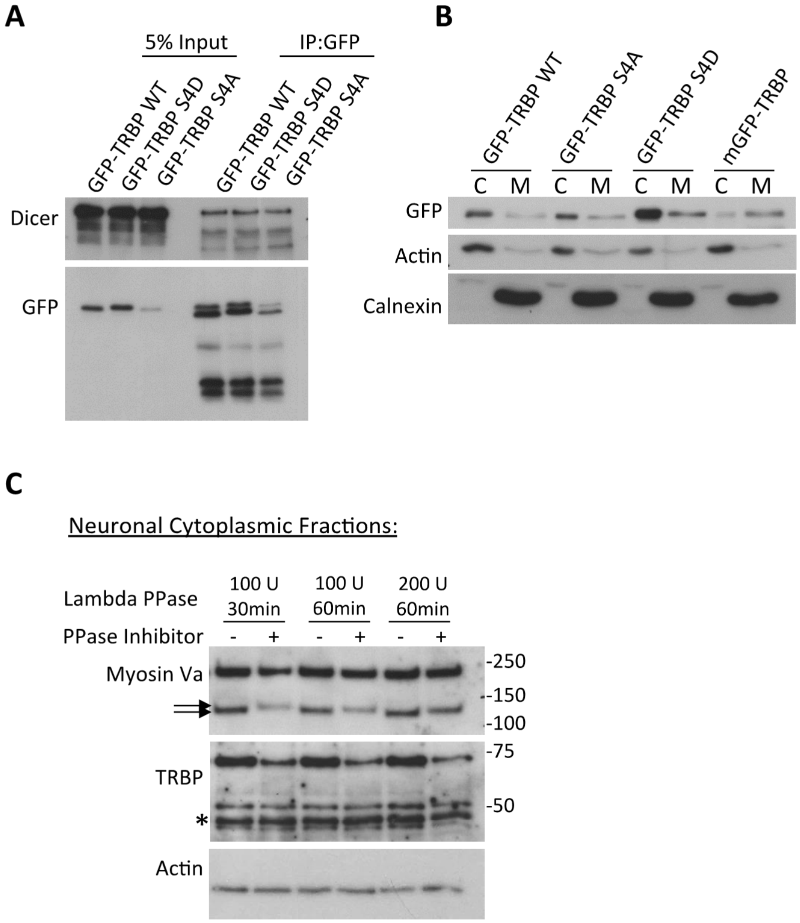

A
5% Input
IP:GFP
GFP-TRBP WT
GFP-TRBP S4D
GFP-TRBP S4A
GFP-TRBP WT
GFP-TRBP S4D
GFP-TRBP S4A
Dicer
GFP
B
GFP-TRBP WT
GFP-TRBP S4A
GFP-TRBP S4D
mGFP-TRBP
C M C M C M C M
GFP
Actin
Calnexin
C
Neuronal Cytoplasmic Fractions:
Lambda PPase
100 U 30min
100 U 60min
200 U 60min
PPase Inhibitor
- + - + - +
Myosin Va
-250
-150
-100
-75
TRBP
-50
*
Actin

Figure S4

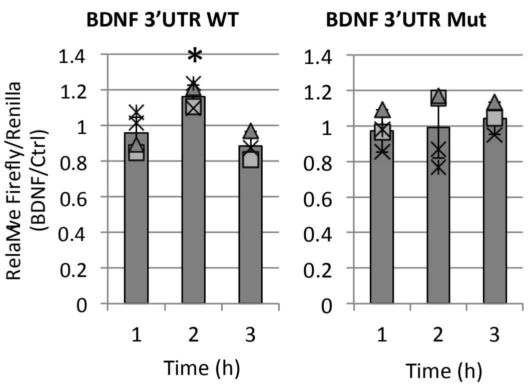

BDNF 3'UTR WT
BDNF 3'UTR Mut
RelaMve Firefly/Renilla (BDNF/Ctrl)
1.4
1.2
1
0.8
0.6
0.4
0.2
0
*
1
2
3
Time (h)
Time (h)

Appendix Figure Legends

Figure S1: Validation of fractionation and imaging results

A) The majority of TRBP is present in the microsomal pellet. Cortical neurons were lysed and subjected to differential centrifugation to isolate the nuclear pellet (P1), the mitochondrial pellet (P2) and ER microsomes (P3). **B)** GW182 does not overlap with the ER marker mCherry-CLIMP63. Hippocampal neurons were transfected with HA-tagged GW182 and mCherry-CLIMP63 for three days and immunostained with anti-HA antibody at DIV 10 (*scale bars; 10µm).* **C&D)** The vast majority of TRBP is present in the cytoplasmic fraction of primary astrocytes **(C)** and HEK293T cells **(D)**, isolated using sequential detergent extraction.

Figure S2: Representative images and raw colocalization values between TRBP and ER markers.

A) Raw co-localization values for TRBP and Calnexin obtained using Pearson's or Mander's co-localization analysis. Individual data points represent the average colocalization values for approximately ten neurons in four independent experiments *(error bars are s.d.).* **B)** Representative images of hippocampal neurons used co-localization analysis. Neurons were transfected with GFP and mCherry-Climp63 and immunostained with antibodies for TRBP, PACT or Ago2 as indicated. GFP was used as a morphological marker. Arrows point to overlapping puncta in the cell soma *(scale bars; 10µm).* **C)** Raw values for TRBP and mCherry-Climp63 co-localization obtained using Pearson's and Mander's co-localization analysis. Individual data points represent the average colocalization values for approximately ten neurons in four independent experiments (*error bars are s.d.).*

Figure S3: TRBP phosphorylation does not affect its sub-cellular distribution or binding to Dicer

A) TRBP phosphorylation does not affect the Dicer-TRBP interaction. HEK293T cells were transfected with GFP-tagged TRBP wild type (WT) or phosphomimetic (S4D) and phosphodeficient (S4A) mutants and coIP was performed using anti-

GFP antibody. **B)** GFP-TRBP S4A and S4D have a similar cytoplasmic distribution as wild-type TRBP. HEK293T cells were transfected with GFP-tagged TRBP versions as indicated and cells were fractionated using sequential detergent extraction. **C)** Cytoplasmic TRBP is not phosphorylated in neurons. Cytoplasmic fractions from cortical neurons were isolated using digitonin and treated with Lambda phosphatase (PPase) (P0753S, NEB) with or without addition of *PhosSTOP* phosphatase inhibitor. Myosin Va was used as a positive control and actin was used as a loading control. The asterisk depicts the expected size for TRBP. The arrows depict the band of myosin Va that responds to phosphatase treatment.

Figure S4: BDNF stimulation leads to a transient increase in the expression of the BDNF 3'UTR dual-luciferase reporter (BDNF 3'UTR WT), but not the miR-16-5p-binding site mutant (BDNF 3'UTR Mut).

Transfected cortical neurons were stimulated with BDNF for 20 minutes and lysed 1-3 hours after stimulation, as indicated on the x-axis ($p=0.02$, $n=3-4$; t-test type 3, error bars are *s.d.).*

<u>Appendix Table S1</u>: Functional annotation clustering of miR-22-3p and miR-9a-3p isomiRs. Shown are the most significant hits from each annotation cluster. Clusters that are significant after multiple comparisons (Benjamini p value) are in italic.

isomiR (3p)	Fold change (log2)/ P-value	Functional Annotation Clustering	Count	P-value	Benjamini
**miR-22 '0'**	+0.005/ p=0.887	Endosome	7	0.0041	0.44
		Protein kinase activity	9	0.00045	0.1
		Positive regulator of cell proliferation	8	0.034	1
		PDZ domain	3	0.049	0.92
		Transmembrane region	24	0.031	0.87
**miR-22 '-1'**	+0.647/ *p=0.014*	*Phosphatase activity*	*6*	*0.00016*	*0.066*
		Pleckstrin homology-like domain	15	0.0062	0.74
		Cell division	8	0.025	1
		Axon guidance	6	0.04	0.92
		HECT	4	0.0089	0.69
		Dendrite	14	0.041	0.92
		Sterile alpha motif/pointed domain	6	0.031	0.92
		Lysosome	6	0.038	0.97
		Cholinergic synapse	6	0.024	0.99
		Regulation of actin cytoskeleton	8	0.041	0.85
**miR-9a '0'**	-0.114/ p=0.303	Myc-type, basic helix-loop-helix (bHLH)	8	0.00098	0.43
		BTB/POZ-like	9	0.0045	0.48
		Peptide alpha-N-acetyltransferase	4	0.0004	0.072
		Positive regulation of transcription	17	0.0038	0.61
		Ribosomal protein import into nucleus	3	0.0035	0.64

		Cellular response to hypoxia	6	0.035	0.87
		Sterile alpha motif domain	5	0.033	0.86
		Keratinocyte differentiation	6	0.0012	0.57
		Non-canonical Wnt-signaling pathway	3	0.034	0.88
		Melanogenesis	5	0.044	0.98
		Proteinase inhibitor, propeptide	3	0.02	0.73
		Magnesium ion binding	9	0.0091	0.41
		Lipid moiety-binding region: S-palmitoyl cysteine	6	0.042	1
		Lambda repressor-like, DNA-binding domain	3	0.047	0.92
**miR-9a '+1'**	-0.725/ *p=0.024*	*Endocytosis*<br>*Endosome*	*12*<br>*10*	*0.00012*<br>*0.00046*	*0.02*<br>*0.042*
		FERM domain	5	0.00083	0.32
		Angiogenesis	5	0.0021	0.12
		Protein kinase-like domain	14	0.0018	0.25
		Cell junction	11	0.011	0.34
		Hepatitis B	6	0.013	0.5
		Lipid degradation	4	0.031	0.36
		Zinc	19	0.036	0.36
