# Supplementary material for: The dynamic recruitment of TRBP to neuronal membranes mediates dendritogenesis during development
Source: EMBO Rep. 2017 Dec 20;19(3):e44853. doi: 10.15252/embr.201744853 (PMC5835843; doi:10.15252/embr.201744853)
Supplement: Supplementary file 2 — Expanded View Figures PDF [file EMBR-19-e44853-s002.pdf]

Expanded View Figures

Figure EV1. Validation of subcellular fractionation and immunocytochemistry.

A RLC proteins are present at ER microsomes. Lysates from 7 DIV cortical neurons were subjected to differential centrifugation to isolate nuclear pellet (P1), postnuclear supernatant (PNS), mitochondrial pellet (P2), and ER microsomal pellet (P3), as depicted in the graphic on the right.

B GFP-tagged TRBP and PACT overlap with the ER marker calnexin. Arrows point to overlapping regions in the cell soma. Hippocampal neurons were transfected at 7 DIV with dsRed, which was used as a morphological marker, and immunostained with anti-calnexin antibody 3 days later (scale bars, 10μm).

C Hippocampal neurons were incubated with Mitotracker Deep Red (500 nM, 15 min) before fixation and immunostaining with TRBP or PACT antibodies. GFP was used as a morphological marker. Box insets represent magnified perinuclear regions (scale bars; 10 μm).

D TRBP-containing RLCs associate at membrane fractions in 7 DIV cortical neurons. Membrane fractions were isolated using sequential detergent extraction, and co-IP was performed using IgG control (IgG-Ctrl) or 2 and 5 μg monoclonal anti-TRBP antibody. The position of the stars corresponds to the correctly sized bands of the depicted proteins (TRBP; ~43 kDa, Ago2; ~97 kDa).

E Total levels of RLC proteins do not change following 20-min BDNF stimulation. Cortical neurons were treated with BDNF or vehicle control, and lysates were processed in Western blot. BDNF stimulation was confirmed using an antibody against phosphorylated TrkA receptor (pTrkA). For quantification, relative band intensities were normalized to actin (n = 4, t-test type 3, error bars are s.d.).

F BDNF stimulation decreases the Pearson's co-localization of TRBP and calnexin at the soma (*P = 0.01) and dendrites (**P = 0.008) of hippocampal neurons. Average co-localization values were normalized to control stimulation in each experiment (n = 4, t-test type 3, error bars are s.d.).

G BDNF stimulation decreases the co-localization of mCherry-Climp63 and TRBP, but not with PACT or Ago2, analyzed using Pearson's correlation (*P = 0.01, n = 4, t-test type 3, error bars are s.d.).

H BAPTA-AM does not change the total levels of Dicer and TRBP. Primary cortical neurons were stimulated with vehicle controls, BAPTA-AM or BDNF as indicated, and lysates were processed in Western blot. Actin was used as a loading control (n = 3, t-test type 3, error bars are s.d.).

© 2017 The Authors

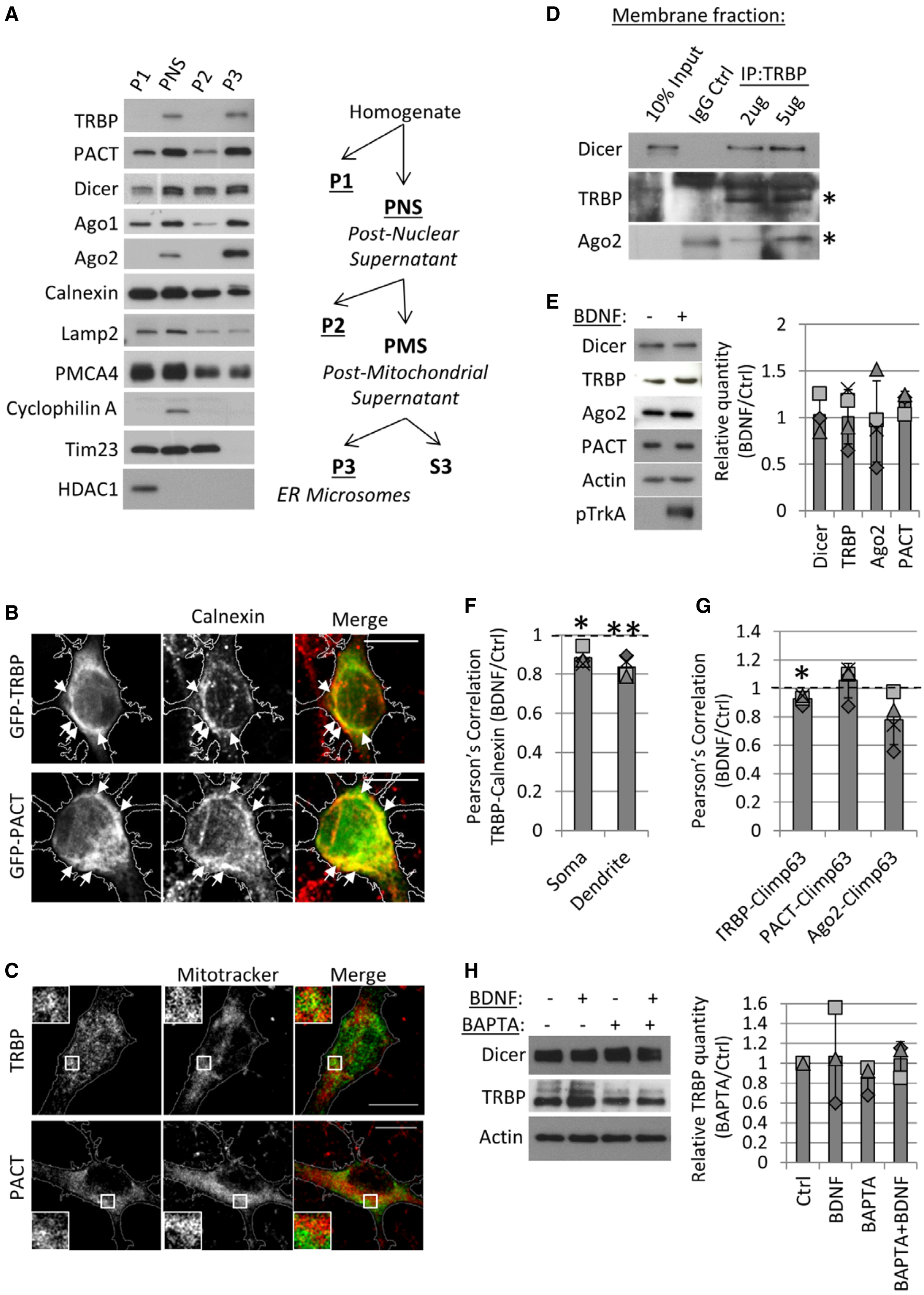

Figure EV1.

© 2017 The Authors

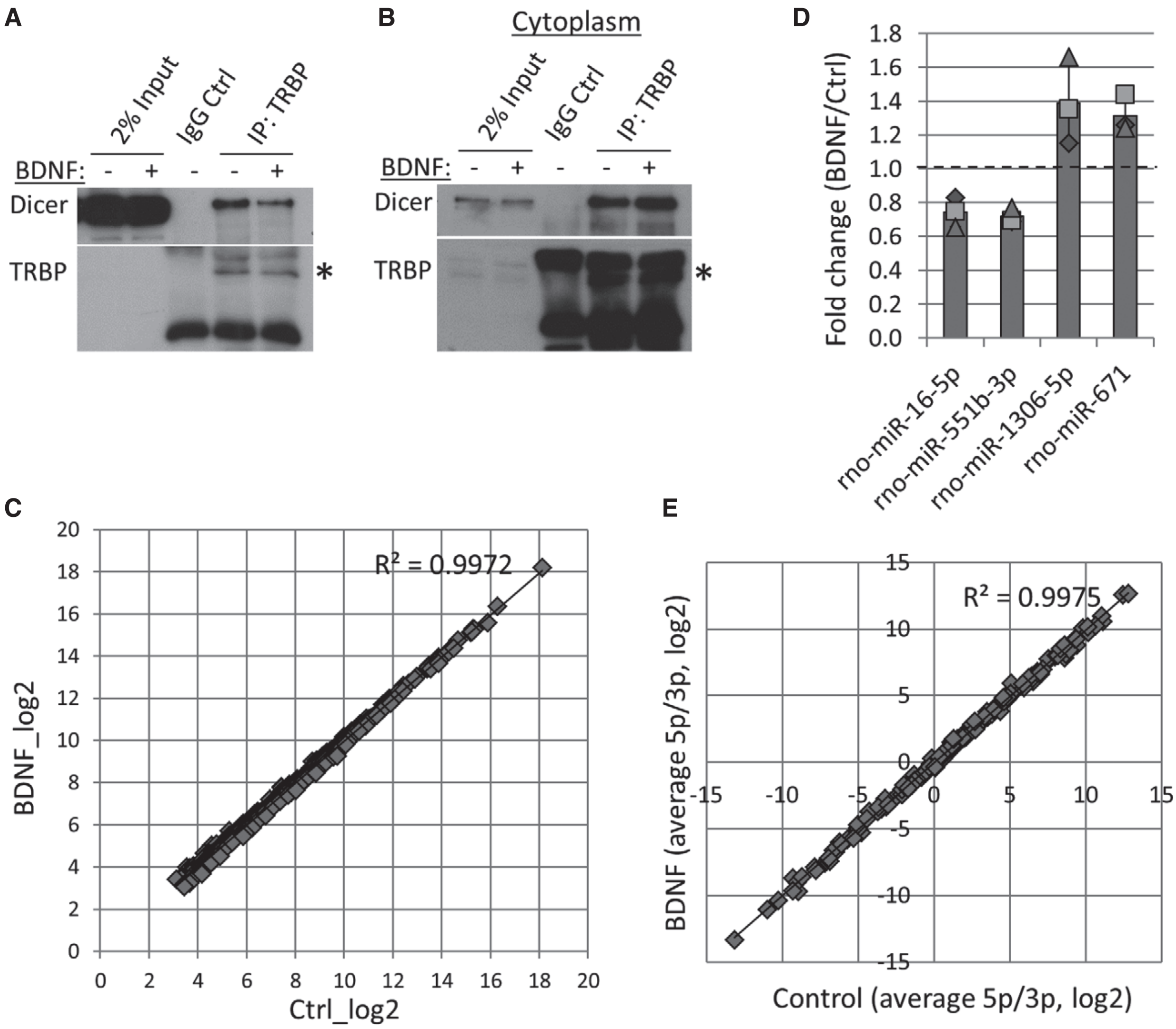

Figure EV2. BDNF affects Dicer binding to TRBP and PACT and bi-directionally regulates the total levels of a few miRNAs.

A BDNF causes a decrease in the Dicer–TRBP interaction in co-IP experiments using a monoclonal mouse TRBP antibody or IgG control. Young cortical neurons were treated with BDNF or vehicle control for 20 min before lysis. The star depicts the right band for immunoprecipitated TRBP (43 kDa).

B BDNF does not affect the interaction of cytoplasmic Dicer and TRBP. Cytoplasmic fractions of stimulated cortical neurons were isolated, and co-IP was performed using anti-TRBP antibody or control IgG. The star depicts the right band for immunoprecipitated TRBP (43 kDa).

C BDNF does not affect global levels of microRNAs in small RNA sequencing. The average read counts (log_2) of miRNAs from three independent experiments were plotted on the *x*-axis for the control and *y*-axis for the BDNF-treated conditions.

D BDNF stimulation bi-directionally regulates the levels of a few microRNAs in small RNA sequencing ($P < 0.05$, $n = 3$, *t*-test type 2, error bars are s.d.).

E BDNF does not lead to changes in 5p versus 3p strand selection. The read count ratio (log_2) of 5p and 3p miRNAs, generated from the same precursor, was calculated from small RNA sequencing data. The averaged values for three independent experiments were plotted on the *x*-axis for the control and *y*-axis for the BDNF-treated conditions.

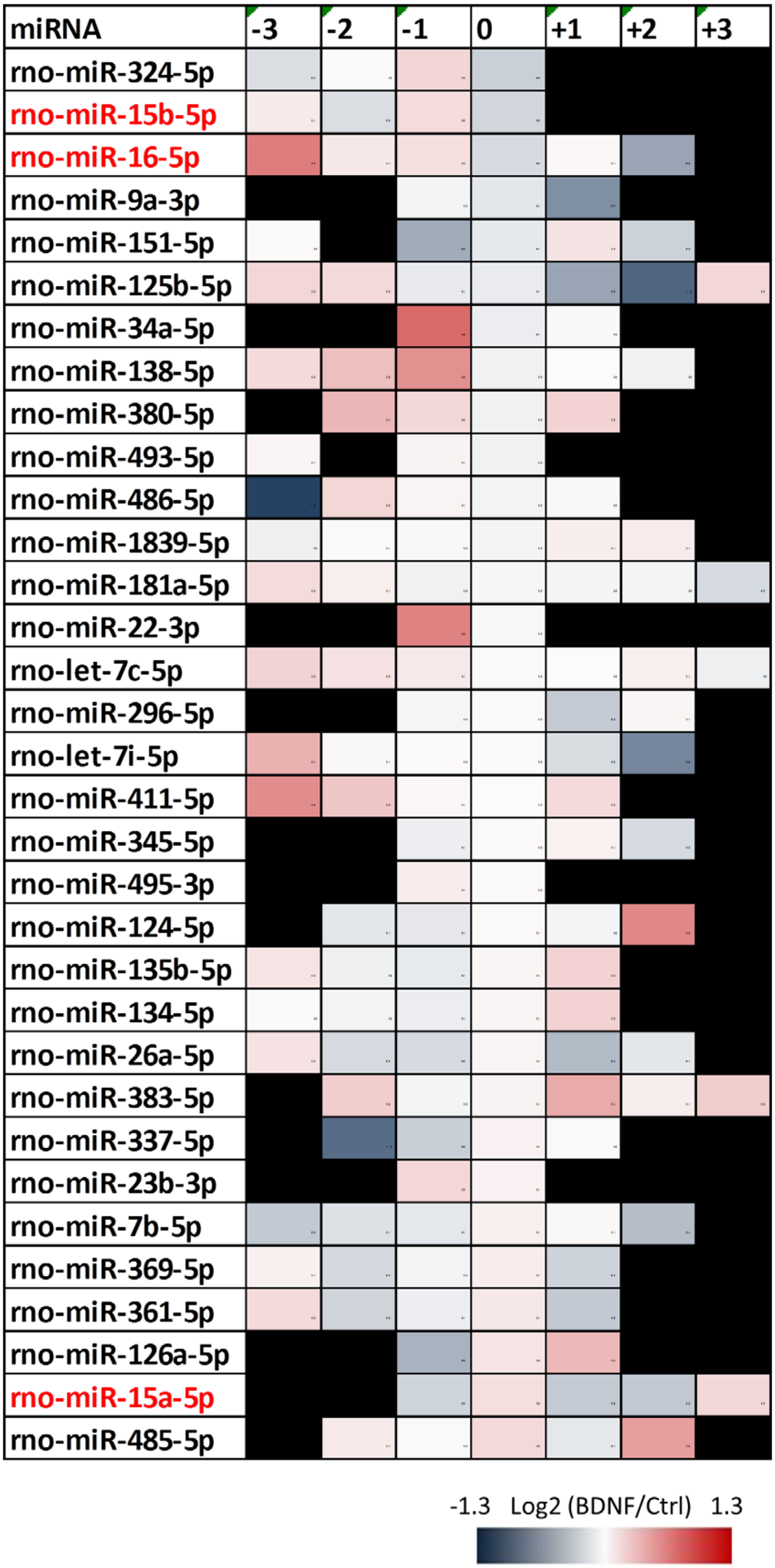

Figure EV3. Short-term BDNF stimulation causes changes in the isomiR levels of many miRNAs.

Cortical neurons were treated with BDNF or vehicle control for 20 min, and isomiR levels were analyzed in small RNA sequencing. Shown are the miRNAs that have a significant change in the relative levels of at least one isomiR. The list is arranged in ascending order of the fold change of canonical isomiRs for each miRNA. Shown in red are members of the miR16 family, miR-16-5P, miR-15b-5p, and miR-15a-5p. Canonical isomiRs for each miRNA are depicted as “0”. Numbers represent the trimming (negative) or addition (positive) of nucleotides to the 3′- or 5′-end of the canonical 5p or 3p isomiRs, respectively. Only templated nucleotide additions and trimmings, which might reflect Dicer-mediated cleavage, have been considered for analysis ($P < 0.05$, $n = 3$, t-test type 3).

 © 2017 The Authors

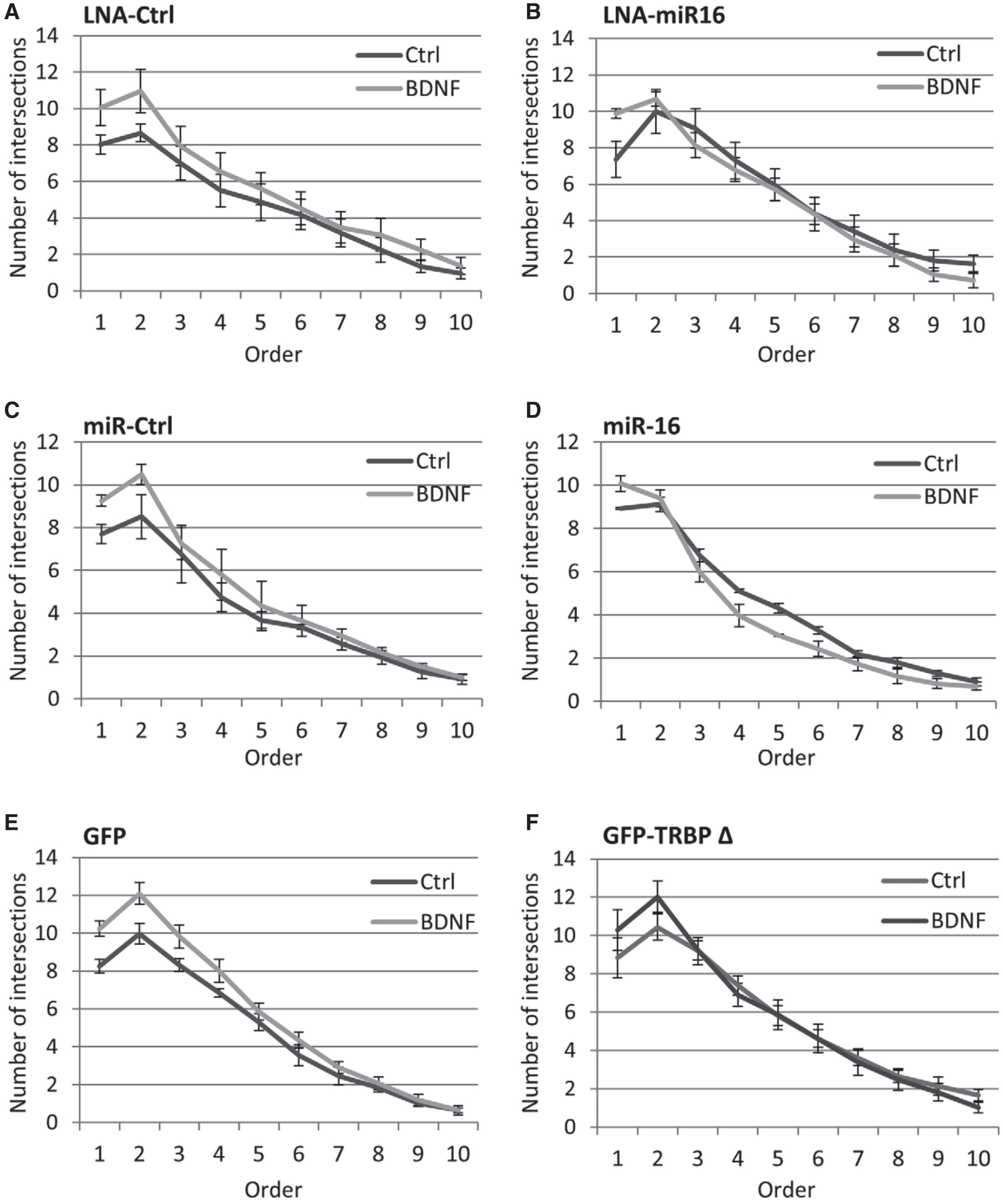

Figure EV4. Sholl analysis in BDNF- or control-treated hippocampal neurons.

A, B Inhibition of miR-16-5p activity using LNA inhibitor (LNA-miR16) occludes BDNF-induced dendritogenesis of hippocampal neurons, compared to control LNA (LNA-Ctrl). The average number of dendritic intersections is shown for each concentric circle in increasing order of distance from the cell soma (n = 3, error bars represent s.e.m.).

C, D Overexpression of miR-16-5p blocks BDNF-induced dendritogenesis compared to a non-functional control miRNA (miR-Ctrl) (n = 4, error bars; s.e.m.).

E, F Overexpression of the Dicer binding-deficient mutant of TRBP (GFP-TRBP Δ) occludes BDNF-induced dendritogenesis of hippocampal neurons compared to GFP control (n = 5, error bars; s.e.m.).

© 2017 The Authors

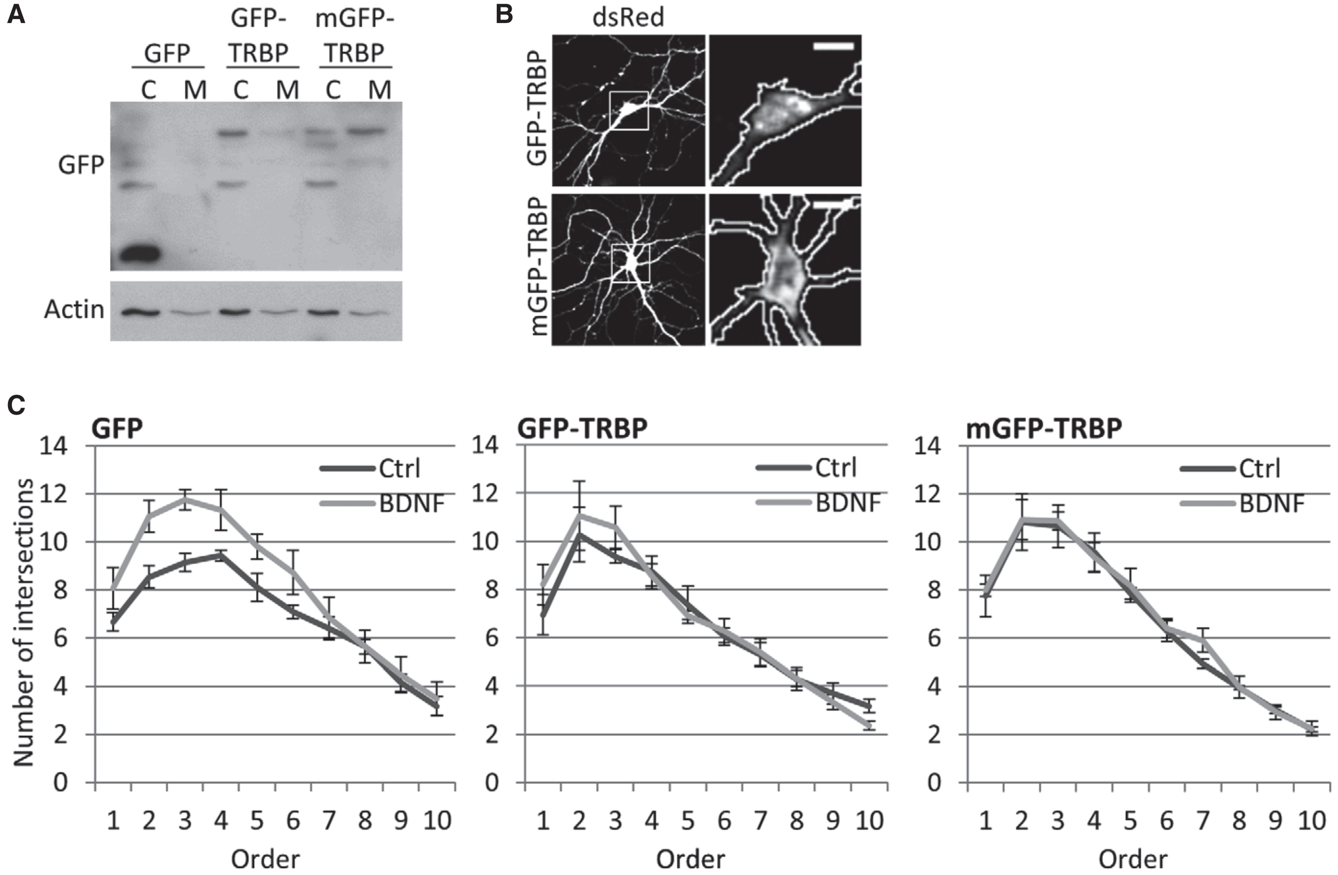

Figure EV5. TRBP relocalization from the membrane to the cytoplasm is required for BDNF-induced dendritogenesis.

A Membrane-targeted GFP-TRBP (mGFP-TRBP) is present at the membrane fraction. HEK293T cells were transfected with 200 ng GFP, 400 ng GFP-TRBP, or 400 ng mGFP-TRBP, and sequential detergent extraction was performed 24 h later to isolate cytoplasmic and membrane fractions.

B Distribution of cytoplasmic and membrane-associated GFP-TRBP in immunofluorescence of hippocampal neurons. Neurons were transfected at 4 DIV with dsRed and GFP-TRBP or mGFP-TRBP and fixed 3 days later. Note that GFP-TRBP is not exclusively cytoplasmic (scale bars are 10 μm).

C Sholl analysis in BDNF- or control-treated hippocampal neurons expressing GFP, GFP-TRBP, or mGFP-TRBP. mGFP-TRBP blocks BDNF-induced dendritogenesis, whereas GFP-TRBP has a partial effect. The number of dendritic intersections is shown for each concentric circle, in increasing order of distance from the cell soma (*n* = 3, error bars; s.e.m.).

 © 2017 The Authors
